# The association of parenting practices with toddlers’ dietary intake and BMI, and the moderating role of general parenting and child temperament

**DOI:** 10.1017/S136898002000021X

**Published:** 2020-10

**Authors:** Jessica S Gubbels, Sanne MPL Gerards, Stef PJ Kremers

**Affiliations:** Department of Health Promotion, Faculty of Health, Medicine and Life Sciences, NUTRIM School of Nutrition and Translational Research in Metabolism, Maastricht University, PO Box 616, 6200 MD Maastricht, The Netherlands

**Keywords:** Parenting practices, Feeding practices, General parenting, Temperament, Dietary intake, BMI, Overweight

## Abstract

**Objective:**

The objective was to examine the association between parenting practices, toddler’s dietary intake and BMI. In addition, potential moderation of these associations by general parenting and child temperament was examined.

**Design:**

The current cross-sectional study assessed parenting practices using the Comprehensive Feeding Practices Questionnaire, general parenting using the Comprehensive General Parenting Questionnaire, child temperament using the Child Behavior Check List, and children’s dietary intake through parental questionnaires. Children’s weight and length were objectively measured to determine BMI *z*-scores. Associations were examined using multiple linear regression analyses. Moderation was examined using interaction terms.

**Setting:**

Home setting.

**Participants:**

393 Dutch toddlers (age 1–3 years) and their parents recruited through fifty childcare centres and preschools in the Netherlands.

**Results:**

Various practices were related to children’s diet and BMI. For instance, the availability of healthy foods is the most important predictor of healthy dietary intake (e.g. *β* = –0·35 for sweets; *β* = 0·18 for fruit). The association of availability with a healthier diet was strongest when parents scored low on the positive parenting style dimensions, including nurturance, structure and/or behavioural control. In addition, it seemed that a high availability of healthy foods and low availability of unhealthy foods is especially beneficial for children showing withdrawal/depressive, anxious or overactive behaviour, while encouraging balance and variety is not beneficial for these children. All other practices were related to children’s diet and/or BMI as well.

**Conclusions:**

The findings underline the importance of viewing the impact of parenting practices in the context of general parenting and child temperament.

Parents play a crucial rule in the development of young children’s eating habits. Specifically, their diet-related parenting practices, also referred to as feeding practices, seem to have an important influence on children’s diet and, consequently, their weight status^(1)^. Parenting practices are content-specific acts of parenting^(2)^, in this case, referring to parenting with regard to the children’s diet^(1)^. Examples of diet-related parenting practices are restriction of intake, pressure to eat and modelling of behaviour^(3,4)^.

There is a lot of evidence regarding the effects of parenting practices on children’s diet (e.g. Refs. (4–6)). Generally, positive approaches seem most promising, while highly controlling practices might be counterproductive^(1,6,7)^. With regard to the effects on very young children (below the age of 5), a review of studies has shown that rewarding with verbal praise might be specifically effective among young children^(5)^. Encouragement to try rather than pressure to eat is associated with a favourable dietary intake^(1,8–10)^ and a decreased BMI^(11)^. Structure-based strategies, such as the availability of foods, modelling and monitoring, are also associated with a healthy intake in young children^(8,9,12)^, and might be even more important than autonomy promoting and controlling practices^(12)^. Non-directive practices (using enhanced availability, education and less discipline practices) were associated with a higher fruit and vegetable intake^(13)^, while an involvement in food preparation was associated with an increased vegetable intake^(9)^. Findings regarding restriction are mixed: restriction has been reported to be associated with both favourable (lower sweet and savoury snack, less soft drink and higher fruit and vegetable intakes^(14)^) and unfavourable (lower vegetable intake^(9)^) dietary intakes in young children. Pressure to eat at age 1 predicted lower fruit consumption at age 2^(15)^. Instrumental feeding (using food as a reward) was associated with unhealthy beverage intake^(10)^.

However, parenting practices do not operate in isolation: their effects are determined by contextual factors^(16)^. Ignoring potential moderators could lead to wrong conclusions and ineffective intervention strategies. Various authors have proposed that more distal general parenting moderates the effect of specific, proximal parenting practices^(2,3,16,17)^. Research seems to indicate that this so-called higher-order moderation^(18)^ is indeed the case^(17)^. General parenting^(2)^ is the emotional climate of the parent–child relationship and is usually measured along dimensions of nurturance/warmth, control/demandingness and structure^(19)^. Restriction was found to be associated with a favourable intake by older children and adolescents, if parents showed a general parenting style characterised by a combination of high nurturance and control^(20–22)^, or high nurturance only^(23)^. Healthy parenting practices had stronger positive effects when used within a positive general parenting context^(24,25)^. Availability had the strongest effect when parents scored high on control but lower on nurturance^(22)^. Finally, modelling was found to be most effective when situated in a highly controlling general parenting style^(26)^. To our knowledge, no studies regarding moderation of diet-related parenting practices by general parenting have been conducted among children below the age of 5.

Furthermore, there are individual differences between children with regard to their response to certain practices: what works for one child might not work for another^(3,5)^. In other words, child characteristics moderate the effects of parenting practices as well. We previously showed that restriction has less favourable or even unfavourable effects on 2-year-olds with a difficult or deviant temperament (e.g. showing depressive, anxious or overactive behaviour)^(14)^. Similarly, Farrow *et al*.^(27)^ have shown that emotional children are less likely to accept parental restriction of food intake. Rollins and colleagues further showed that restriction has particularly unfavourable effects on preschoolers with lower inhibitory control^(28)^.

The current study examines the association between a broad range of diet-related parenting practices, and dietary intake and weight status of very young children (1–3 years old). In addition, the study examines the moderation of these associations by general parenting and child temperament.

## Methods

### Respondents and procedure

Participants were recruited through fifty childcare centres and preschools in the south of the Netherlands (Noord-Brabant and Limburg provinces) for a larger cross-sectional study about the determinants of children’s energy balance-related behaviours and weight status. Prior to the recruitment of parents, these centres were approached by telephone or e-mail; centre managers approved participation. Parents of children aged 1–3 years received a letter or e-mail about the study. In addition, parents were recruited by research assistants at drop-off and pick-up times at childcare centres and preschools. In order to participate, children had to be able to walk independently. In case parents had more than one child going to the childcare centre or preschool, parents were asked to participate with their older child meeting the inclusion criteria. In total, parents of 480 children agreed to participate, and provided written informed consent. In return for their participation, parents received feedback on their children’s behaviour and some general tips for a healthy weight at the end of data collection.

Thirteen children were excluded because of severe food intolerance or allergies (*n* 6), being indicated as too young for the study by their parents (*n* 6) or having a severe physical disability or growth retardation (*n* 1), resulting in a sample of 467 children. Data collection was conducted from November 2014 to January 2016.

### Measures

The study included an online questionnaire for parents and an assessment of child anthropometrics at the childcare centre or preschool. Either parent could fill in the questionnaire.

#### Parenting practices

Parenting practices were assessed using the parental questionnaire. Forty items of the Comprehensive Feeding Practices Questionnaire (CFPQ)^(29)^ were used to assess parents’ food-related parenting practices. The CFPQ consists of questions and statements regarding feeding practices, which are answered using a five-point Likert scale ranging from ‘never’ (1) to ‘always’ (5) for the questions, and from ‘disagree’ (1) to ‘agree’ (5) for the statements. Eight parenting practices were assessed (see Table 1): teaching about nutrition (e.g. ‘I discuss with my child why it’s important to eat healthy foods’), emotion regulation (e.g. ‘How often do you give your child something to eat or drink if he/she is upset, even if you think he/she is not hungry?’), pressure to eat (e.g. ‘My child should always eat all of the food on his/her plate’), restriction of intake (e.g. ‘If my child eats more than usual at a meal, I try to restrict his/her eating at the next meal’), availability (i.e. healthy environment, e.g., ‘Most foods in my house are healthy’), encourage balance and variety (e.g. ‘I encourage my child to try new foods’), child control over eating (e.g. ‘How often do you let your child eat whatever he/she wants?) and modelling of healthy eating (e.g. ‘I try to show enthusiasm about eating healthy foods’).

**Table 1:** Descriptive and scale information of parenting practices, general parenting and child temperament (*N* 393)

	Number of items	Cronbach’s *α*	Mean	sd
Parenting practices*				
Teaching about nutrition	3	0·59	3·49	0·90
Emotion regulation	3	0·69	1·49	0·49
Pressure to eat	4	0·70	3·12	0·86
Restriction of intake	5	0·55	3·26	0·65
Availability	4	0·59	3·62	0·69
Encourage balance and variety	3	0·57	4·34	0·60
Child control over eating	5	0·50	2·49	0·53
Modelling of healthy eating	4	0·73	4·23	0·61
General parenting†				
Nurturance	14	0·78	4·65	0·28
Structure	7	0·78	4·51	0·43
Behavioural control	8	0·84	4·54	0·44
Child temperament‡				
Oppositional	17	0·89	1·43	0·37
Withdrawal/depressive symptoms	10	0·59	1·08	0·18
Anxious	10	0·78	1·32	0·33
Overactive	5	0·66	1·50	0·41

* Parenting practices measured using Comprehensive Feeding Practices Questionnaire (CFPQ) (scale 1–5)^(29)^.

† Parenting styles measured using CGPQ/1–3 (scale 1–5)^(31)^.

‡ Temperament measured using Child Behavior Check List for toddlers (CBCL)/2–3 (scale 1–5)^(32)^.

All selected items of the CFPQ were translated into Dutch by one of the authors (JSG); another author (SPJK) checked the translation. Cronbach’s *α* was calculated to examine reliability of CFPQ scales. Cronbach’s *α* > 0·50 was considered acceptable^(30)^; items were deleted until an acceptable Cronbach’s *α* was reached. For the scale ‘encourage balance and variety’, one item had to be deleted. For all other scales, all items were maintained. An average score of the included items was calculated for each scale. The final scales and their reliability are presented in Table 1.

#### Moderators: general parenting and child temperament

General parenting and child temperament were assessed through the parental questionnaire to examine their moderating role in the relationship between parenting practices and child outcomes.

General parenting was assessed using a selection of twenty-nine items of the Comprehensive General Parenting Questionnaire for 1- to 3-year-olds (CGPQ/1–3)^(31)^, which is a modified version of the original CGPQ^(19)^. The selected items assessed three main scales of CGPQ/1–3: nurturance (e.g. ‘My child and I have warm, affectionate moments together’), structure (e.g. ‘I try not to change the rules at home very often’) and behavioural control (e.g. ‘I have clear expectations for how my child should behave’). All items were answered on a five-point Likert scale from ‘strongly disagree’ (1) to ‘strongly agree’ (5).

The Child Behavior Check List for toddlers (CBCL/2–3)^(32)^ was used to assess children’s temperament. CBCL/2–3 has shown high cross-cultural validity, including in Dutch samples^(32)^. The subscales for oppositional (seventeen items, e.g., ‘My child is stubborn’), withdrawal/depressive (ten items, e.g., ‘My child doesn’t answer when others talk to him/her’), anxious (ten items, e.g., ‘My child is easily upset by new people or situations’) and overactive (five items, e.g., ‘My child cannot sit still’) behaviours were used. For each of the forty-two items, parents could indicate whether the items were ‘not true’ (1), ‘somewhat or sometimes true’ (2) or ‘very true or often true’ (3) for the child. For each of the four scales, an average of the items was calculated.

All selected items of CGPQ/1–3 and CBCL/2–3 were translated into Dutch by one of the authors (JSG); another author (SPJK) checked the translation. In addition, the author of the original questionnaire checked the translations of CGPQ^(19)^. Cronbach’s *α* was calculated to examine the reliability of the scales. No items had to be deleted to reach an acceptable Cronbach’s *α* (>0·50)^(30)^. An average score of the included items was calculated for each scale (see Table 1).

#### Child outcomes

Children’s dietary intake was assessed using an FFQ filled out by the parents. Parents were asked how often their child consumed fruit, vegetables, pastry (e.g. cookies, cake), sweets, savoury snacks, water (including unsweetened tea) and sugary drinks (including processed fruit juices, excluding fresh fruit juice). Answering options were ‘never or less than once a week’, ‘1–3 times a week’, ‘4–6 times a week’, ‘once a day’, ‘twice a day’, and ‘≥3 times a day’. Intake was recoded into weekly intake frequency using the middle of each category comprising a range (e.g. ‘1–3 times a week’ was recoded into two times a week).

Trained research assistants measured children’s height and weight at the childcare centre or preschool during regular opening hours, according to a standardised measuring protocol, using a standard scale and a stadiometer. Children were weighed and measured once without shoes and heavy clothes, and the childcare staff was asked to change children’s diaper, if applicable, before the measurements. For twenty-six children, a valid weight and/or height measurement could not be taken due to a variety of reasons (e.g. the child not being cooperative, being asleep or not being present during the measurement). Measurements of non-cooperative children (e.g. wiggling, refusing to take off shoes) were excluded. Height and weight were used to calculate BMI, which was converted to BMI *z*-scores, reflecting the number of standard deviations the child differed from the age- and sex-specific mean of the national reference population^(33)^.

#### Child and parental background characteristics

Child and parental background characteristics were assessed via the parental questionnaire as potential covariates of the models. Children’s sex and age (in months, derived from birth date and date of completion of the questionnaire) were assessed. Questionnaire completer was assessed by asking who filled out the questionnaire (mother, father or together). In addition, age (in years), country of birth (Netherlands *v*. other), education level and BMI (in kg/m^2^, calculated from self-reported weight and height) of both parents were assessed. Education level was recoded into low (elementary school, lower secondary education, lower vocational education), medium (medium vocational education, higher secondary education and college prep) and high (higher vocational education, university) and then recoded into dummy variables for low and high compared to medium.

### Statistical analyses

All analyses were conducted using SPSS 24.0. *P* < 0·05 was considered statistically significant. Independent *t* tests and *χ* ^2^ tests were used to compare children who were included (those with data regarding parenting and one or more child outcomes) with those who were excluded due to incomplete data. Descriptive statistics were used to examine all variables included in the study. Multiple linear regression analyses were conducted to examine the associations between parenting practices and child outcomes (fruit, vegetable, pastry, sweets, savoury snacks, water and sugary drinks intakes and BMI *z*-score), adjusting for children’s (age and sex) and parents’ (questionnaire completer, and age, country of birth, education level and BMI of both parents) background characteristics.

Next, interaction terms between each of the parenting practices and each potential moderator (each general parenting and child temperament scale) were added to the regression models. The interaction terms were added one by one to the model, and the main effects of the included moderator were added as well. In case an interaction term was significant, the sample was split into two roughly equal-sized groups based on the median of the moderator variable (general parenting or child temperament scale) to examine the association of parenting practices separately for both groups. Only the results of the interaction-based subgroup analyses in which the concerning parenting practice had a significant association with the outcome are presented.

## Results

Data regarding parenting and one or more child outcomes were available for 393 of the 467 participating children (84·2 %). Analyses showed that these included children did not differ significantly from those who were excluded, due to incomplete data, on any of the children’s or parents’ background characteristics. The background characteristics of the included children and their parents, as well as the child outcomes, are presented in Table 2. Children were on average 3 years old. Most questionnaires were filled out by mothers (83·8 %); most questionnaire completers and their partners were born in the Netherlands (95·9 % of completers and 96·3 % of their partners); and most parents were highly educated (64·8 and 60·5 %, respectively).

**Table 2:** Descriptive of background characteristics and outcome variables (*N* 393)

	*n* *	%	Mean	sd
Child				
Sex				
Boy	194	49·4		
Girl	199	50·6		
Age (months)			36·1	18·3
BMI *z*-score				
Underweight†	7	2·1	0·2	0·9
Normal weight‡	270	80·8		
Overweight/obese§	57	17·1		
Dietary intake (times per week)				
Fruit			9·5	4·3
Vegetables			6·8	3·4
Pastry			4·8	3·1
Sweets			4·7	4·3
Savoury snacks			2·7	3·3
Water			11·1	7·8
Sugary beverages			11·3	6·8
Questionnaire completer				
Mother	327	83·8		
Father	41	10·5		
Together	22	5·6		
Age (years)			34·4	4·4
Country of birth				
Netherlands	377	95·9		
Other	16	4·1		
Education level				
Low	18	4·6		
Medium	130	30·6		
High	254	64·8		
BMI			24·3	4·1
Partner				
Age (years)			36·5	4·6
Country of birth				
Netherlands	363	96·3		
Other	14	3·7		
Education level				
Low	34	9·1		
Medium	114	30·4		
High	227	60·5		
BMI			24·9	3·2

* *n* deviates from the total sample size due to missing values; valid percentages are presented.

† Underweight <5th percentile.

‡ Normal weight 5th–85th percentile; overweight/obese >85th percentile.

The mean BMI *z*-score (0·22) of the children was above the average of the reference population^(33)^. Children ate vegetables almost daily (6·8 times a week), and fruit more than once a day (9·5 times a week) on average. They drank water and sweet drinks about equally often (both around eleven times a week on average).

### Associations between parenting practices and children’s dietary intake and BMI *z*-score

Table 3 shows the various bivariate correlations between parenting practices and child outcomes. A high availability of healthy foods and low availability of unhealthy foods was correlated with healthy intake across outcomes (i.e. higher fruit, vegetable and water intakes, and lower pastry, sweets, snacks and sugary beverages intakes). Similarly, modelling of healthy eating was correlated with lower sweets, snacks and sugary beverage intakes, and higher water intake. Emotion regulation and child control over eating were correlated with an increased intake of unhealthy foods – in the case of emotion regulation with a higher BMI *z*-score.

**Table 3:** Correlations between parenting practices and children’s dietary intake and BMI *z*-score

Parenting practices	Correlation coefficient†							
	Fruit	Vegetables	Pastry	Sweets	Savoury snacks	Water	Sugary beverages	BMI *z*-score
Teaching about nutrition	0·12*	0·09	0·00	0·01	–0·05	0·13**	–0·01	0·02
Emotion regulation	–0·06	–0·05	0·21***	0·11*	0·26***	0·00	0·10	0·13*
Pressure to eat	–0·07	0·01	0·11*	0·11*	0·01	–0·05	0·07	–0·12*
Restriction of intake	–0·08	0·03	–0·10	–0·14**	–0·07	0·08	–0·09	0·21***
Availability	0·18***	0·16**	–0·21***	–0·32***	–0·19***	0·21***	–0·22***	–0·01
Encourage balance and variety	0·09	0·06	–0·06	–0·05	–0·06	0·16**	–0·05	0·00
Child control over eating	–0·03	–0·10*	0·06	0·13*	0·18***	0·01	0·17**	–0·02
Modelling of healthy eating	0·06	0·09	–0·04	–0·16**	–0·22***	0·22***	–0·14**	0·06

† Results of bivariate Pearson correlations.

*P < 0·05, **P < 0·01, ***P < 0·001.

Table 4 shows the adjusted associations between parenting practices and child outcomes. In line with the correlations, a high availability of healthy foods and low availability of unhealthy foods was consistently associated with a healthy intake across outcomes. Parental modelling of healthy eating was also associated with a healthy intake (lower savoury snack and higher water intake). On the other side, the use of food to regulate emotions, child control over eating and encouraging balance and variety were associated with a unhealthy intake and a higher BMI *z*-score. Conflicting results were found for teaching about nutrition, which was associated with a higher intake of both fruit and sweets.

**Table 4:** Associations between parenting practices and children’s dietary intake and BMI *z*-score

Parenting practices	Standardised regression coefficient (*β*)†							
	Fruit	Vegetables	Pastry	Sweets	Savoury snacks	Water	Sugary beverages	BMI *z*-score
Teaching about nutrition	0·11*	–	–	13*	–	–	–	–
Emotion regulation	–	–	0·17**	–	0·21***	–	–	0·11*
Pressure to eat	–	–	–	–	–	–	–	–0·13*
Restriction of intake	–	–	–	–	–	–	–	0·23***
Availability	0·16**	0·14**	–0·18**	–0·35***	–	0·14*	–0·21***	–
Encourage balance and variety	–	–	–	–	0·14*	–	–	–
Child control over eating	–	–	–	–	0·11*	–	0·13*	–
Modelling of healthy eating	–	–	–	–	–0·26***	0·13*	–	–

† Results of the final models of backward regression analyses (only showing regression coefficients for the remaining independent variables in each model). All analyses were adjusted for children’s sex and age (in months), questionnaire completer (mother or father), and both parents’ age (in years), country of birth (Netherlands *v.* other), education level (low, medium, high) and BMI.

*P < 0·05, **P < 0·01, ***P < 0·001.

### Interaction between parenting practices and general parenting

There were several interactions between parenting practices and general parenting. Only the results of analyses in which the regression coefficient for parenting practices was statistically significant (*P* < 0·05) in one or both subgroups are presented. The overall beneficial association of availability with dietary outcomes (see Table 4) was moderated by general parenting. Specifically, lower scores on general parenting style dimensions were associated with stronger positive effects of a high availability of healthy foods and low availability of unhealthy foods: food availability was associated with lower pastry (*β* = –0·212, *P* = 0·014) and sweets (*β* = –0·314, *P* < 0·001) intakes when parents scored lower on nurturance, but not in parents who scored higher on nurturance (non-significant). Similarly, the association of having many healthy foods and few unhealthy foods at home showed a stronger negative association with sweets intake for parents who scored lower on structure (*β* = –0·483, *P* < 0·001) and behavioural control (*β* = –0·308, *P* < 0·001) in general parenting style dimensions, compared to parents who scored high on structure (*β* = –0·238, *P* = 0·008) and behavioural control (*β* = –0·294, *P* = 0·002). An exception to this pattern is the association of availability with water intake: this association was present when parents scored high on nurturance (*β* = 0·245, *P* = 0·008), but not when parents scored low on nurturance (non-significant).

In addition to the interactions of availability with general parenting, there was also an interaction between child control over eating and nurturance. Child control over eating was associated with higher sweets intake in children whose parents showed low nurturance (*β* = 0·144, *P* = 0·048). This association was not present when parents showed a high nurturance (non-significant). In all other cases, the interaction between practices and general parenting was non-significant, or the association between practices and outcomes was significant in neither of the moderator subgroups.

### Interaction between parenting practices and child temperament

There were several significant interactions between parenting practices and child temperament. Although food availability showed an overall beneficial association with a broad range of dietary outcomes (see Table 4), a positive association of fruit intake with withdrawal and depressive symptoms (*β* = 0·27, *P* = 0·003), as well as of vegetable intake with anxious behaviour (*β* = 0·34, *P* < 0·001) was observed, but not for other children (non-significant). Encouraging balance and variety had a positive association with vegetable intake in children not showing withdrawal/depressive behaviour (*β* = 0·23, *P* = 0·042), but not in children with withdrawal/depressive behaviour (non-significant). The overall undesirable association of encouraging balance and variety with a higher savoury snack intake (*β* = 0·14, *P* = 0·021; see Table 4) seemed to be explained by the association in overactive children (*β* = 0·37, *P* = 0·001); in other children, this association was not present (non-significant). Furthermore, teaching about nutrition was positively associated with fruit intake in overactive children (*β* = 0·31, *P* = 0·004), but not in others (non-significant). The overall positive association between modelling of healthy behaviour and water intake (*β* = 0·13, *P* = 0·017; see Table 4) seemed to be explained by the association in children not showing withdrawal/depressive behaviour (*β* = 0·24, *P* = 0·046), while this association was not present in other children (non-significant).

In line with its overall undesirable association with dietary intake (see Table 4), emotion regulation using food was associated with a higher BMI *z*-score in overactive children (*β* = 0·20, *P* = 0·031), and with a higher savoury snack intake in non-anxious children (*β* = 0·22, *P* = 0·014). These associations were not present in non-overactive and non-anxious children (both non-significant). Emotion regulation using food was also associated with a lower sugary drink intake in non-anxious children (*β* = –0·18, *P* = 0·047), but not in anxious children. Child control over intake was associated with a higher water intake in overactive children (*β* = 0·19, *P* = 0·046), but not in non-overactive children (non-significant). Restriction was associated with a decreased pastry intake in anxious children (*β* = –0·20, *P* = 0·018), but not in other children (non-significant).

## Discussion

The current study examined the association between diet-related parenting practices and dietary intake and BMI *z*-scores of very young children. Overall, effect sizes were rather small (all standardised regression coefficients from the main analyses ≤0·35), although several were statistically significant and some revealed a consistent pattern. Furthermore, small effects at a young age can have a large impact over time, as dietary habits are often established at a young age and can track into later ages (e.g. Ref. (34)). Most notable is the consistent favourable association of having many healthy and few unhealthy foods available with almost every outcome across the dietary intake spectra. Moderation analyses indicated that healthy food availability at home is especially important for children who are raised by parents with suboptimal general parenting styles, as well as for children with a more problematic temperament.

The consistent association of availability of healthy foods (fruit, vegetables and water) with less intake of unhealthy foods (pastry, sweets and sugary beverages) is not surprising. Various literature reviews have consistently indicated the availability of foods as one of the most important and strongest predictors of children’s dietary intake^(5–7)^. Our results further showed that food availability is especially beneficial for children with a more problematic temperament. Although not assessed specifically in the current study, previous research suggests that accessibility, in addition to availability, is also very important^(7)^. An example of making healthy foods more accessible to young children is pre-cutting and peeling fruits and vegetables^(35)^. Furthermore, we found that modelling of healthy intake was associated with a lower savoury snack intake and a higher water intake, in line with previous research^(5)^. Taylor and colleagues have argued that structure-based feeding strategies, including availability and modelling but also monitoring, might be more important than any explicit rules about food^(12)^, which our findings seem to underline. Furthermore, as expected, using foods to regulate emotions was associated with an increased pastry and savoury snack intake and a higher BMI *z*-score. In line with this, previous research has shown that using foods to regulate emotions increased children’s preference for high-fat and sugar foods^(36)^, eating in the absence of hunger^(37)^, emotional eating^(38)^ and excessive weight^(39)^.

Pressure to eat was associated with a lower BMI *z*-score, while a restriction of intake was associated with a higher BMI *z*-score. Increased liking and intake of restricted foods has been often, though not consistently, reported^(5)^, which could lead to an increased BMI^(40)^. Similarly, some, but not all, studies have indicated a decreased intake of pressured foods, especially among younger children^(5)^, perhaps consequently decreasing BMI. However, as the current study had a cross-sectional design, these findings could also be a result of reverse causation, in which case pressure and restriction do not (only) lead to, respectively, lower and higher BMI, but are parents’ reaction to these BMI^(41,42)^. In fact, both mechanisms probably occur^(41,42)^, with parents and children getting trapped in a negative spiral. In the case of restriction, this means that parents exert increased restriction in response to a higher BMI, in turn leading to an even higher BMI^(41)^. With pressure, the opposite occurs: parents pressure a thinner child to eat more, resulting in even further decreasing BMI^(42)^.

Unexpectedly, teaching children about nutrition was associated with an increased sweets intake (but not any other dietary intake variables). Based on a literature review, Yee *et al*.^(5)^ have argued that education might be more effective for healthy food than unhealthy food, such as sweets. Nonetheless, a counterproductive effect is not to be expected. The developmental stage might also be important, as education seems more effective in older children^(5)^. At 1–3 years of age, the current sample might be too young to understand educational messages about nutrition. More research will be needed to further examine the appropriateness of educational strategies at different developmental stages^(5)^. Furthermore, it is important to note that the bivariate correlation analyses did not reveal this unexpected association between teaching and sweets intake, in addition to some other differences between the bivariate and adjusted analyses. This indicates the importance of looking at parenting practices within the context of other practices and parent and child characteristics. In line with the findings regarding teaching about nutrition, age might have played a role in the finding that child control over intake was associated with an increased intake of savoury snacks and sugary beverages. Previous research has indicated that young children might have a poor regulation of energy intake and are primarily responsive to environmental stimuli such as availability^(43)^. This is in line with our findings regarding child control and availability of foods. Granting young children large autonomy over their intake might not be suitable at a young age. In the general parenting literature, this is called scaffolding: exposing children to age- and developmental stage-appropriate activities, providing just enough structure and assistance to help them^(19)^. Large control of a toddler over his/her intake might just be a bridge too far.

General parenting moderated the association of several practices with diet. The association of food availability with a healthier diet was strongest when parents scored low on the positive general parenting style dimensions including nurturance, structure and/or behavioural control. In addition, child control over eating was associated with an increased sweets intake when parental nurturance was low. Based on these findings, we hypothesised that while the impact of overt practices seems optimised within a positive general parenting style with high nurturance and behavioural control^(5)^, covert or structure-based practices might have a stronger impact on children raised in families with a less desirable general parenting style. More research is, however, needed to further examine this hypothesis. The fact that parenting practices interacted with general parenting is in line with our current understanding that parenting practices are part of a complex interactive family system, in which multiple levels of influence interact, as proposed in the LIFES framework^(16)^. If viewed in isolation of this context, conclusions about the effects of parenting practices are perhaps wrong, and our intervention efforts based on these conclusions consequently a waste of time and money.

The majority of interactions between parenting practices and child temperament tested were non-significant. Nonetheless, a number of child temperament scales moderated the association between practices and outcomes. Overall, it seemed that a high availability of healthy foods and low availability of unhealthy foods was especially beneficial for children with a more difficult temperament (i.e. showing withdrawal, anxious or overactive behaviours), while encouraging balance and variety was not beneficial, or was even counterproductive, for these children. Children with a more difficult temperament might thus need more structure-based, covert instead of overt strategies. This is in line with our previous finding that 2-year-olds with a deviant temperament responded less well to restriction^(14)^, although we were not able to replicate this finding in the current study with regard to restriction specifically. Furthermore, there were also some interactions contradicting this hypothesis, instigating further research. In addition to the current findings, other potential moderators of parenting also need to be examined. Food responsiveness of the child, for instance, seems to be another important moderator of effects of parenting practices^(44)^. It is important to realise that when it comes to parenting, there is no ‘one-size-that-fits-all’^(45)^. In addition, it would be interesting to examine the three-way interaction between general parenting, parenting practices and child temperament, as general parenting and child characteristics also seem to interact with each other^(46)^. The current sample size did not permit an examination of such complex three-way interactions, however.

The current study has several strengths and limitations. Strengths include the young age of the sample and the use of validated measures for parenting practices^(29)^, general parenting^(31)^ and child temperament^(32)^, although the translated Dutch versions of CGPQ and CFPQ were not validated, and Cronbach’s *α* of some of the practice scales could be considered moderately low, though acceptable, according to Portney and Watkins^(30)^. Another strength is that children’s BMI was objectively measured, although duplicate measures were not taken and intra- and inter-rater reliability could, therefore, not be estimated. Furthermore, we used a national reference population for BMI, although this sample was somewhat dated^(33)^. The main limitation of the current study is the cross-sectional design, limiting inferences about causality. Furthermore, children’s dietary intake was parent-reported, potentially causing bias, and regarded intake frequency, but the amount consumed per eating occasion was not registered. There was little variation in general parenting and temperament scales, potentially caused by social desirability. A different measure of child temperament might have been more appropriate. Finally, the sample was relatively highly educated and predominantly Dutch native, but was too small to examine the potential three-way interactions between parenting practices, general parenting and child temperament. Longitudinal research with a very large sample, with more accurate assessments of dietary intakes, is advised to further disentangle the complex interactions between the parent and children.

Overall, we conclude that several parenting practices are important for shaping toddlers’ diet and weight status, especially the availability of foods at home and modelling. Furthermore, the interactions with general parenting and child temperament clearly show that these practices cannot be viewed in isolation, but need to be regarded within the context of a broader ecological system^(18)^.
